# Women’s visibility at European Society for Clinical Nutrition and Metabolism congresses from 2011 to 2019: Is the floor yours?

**DOI:** 10.3389/fnut.2022.963577

**Published:** 2022-11-17

**Authors:** Emilie Occhiali, Zoe Demailly, Thomas Clavier, Najate Achamrah

**Affiliations:** ^1^Department of Anaesthesiology, Critical Care and Perioperative Medicine, Rouen University Hospital, Rouen, France; ^2^Department of Nutrition, Rouen University Hospital, Rouen, France; ^3^Clinical Investigation Center CIC 1404, INSERM, Rouen University Hospital, Rouen, France

**Keywords:** gender, women, visibility, ESPEN, congress, nutrition

## Abstract

**Purpose:**

Participating in international conferences is an essential way to promote scholarly work. We aimed to assess the trend of women’s visibility at the European Society for Clinical Nutrition and Metabolism (ESPEN) congress by describing the evolution of the proportion of women speakers between 2011 and 2019.

**Materials and methods:**

This is a retrospective study including public data obtained from the 2011, 2015, and 2019 ESPEN congresses. The primary endpoint was the percentage of women speakers in major oral sessions (oral communications and specific conferences including prestigious lectures). The secondary endpoints were the proportion of women in other high-visibility positions (moderators, industry-led symposia interventions) and countries of origin.

**Results:**

The proportion of women speakers in oral communications remained stable between 2011 and 2019 [43% (43/100) vs. 41% (46/111), respectively; *p* = 0.89]. The proportion of women moderators in oral communications sessions significantly increased between 2011 and 2019 [13% (6/45) vs. 41% (19/46), respectively; *p* = 0.004]. The percentage of women speakers and moderators in industry-led symposia significantly increased between 2011 and 2019 [11% (2/18) vs. 41% (11/27), *p* = 0.05; 0% (0/6) vs. 60% (6/10), *p* = 0.03, respectively]. The percentage of women moderators in educational sessions also remained stable during the period with a marked under-representation of women in 2015. During all three congresses, women from the host countries were over-represented as moderators compared to women from other countries.

**Conclusion:**

The percentage of women speakers in oral communications remained stable in the last 8 years at ESPEN congresses, although women’s representation in other high-visibility positions has increased. As men remained over-represented, women should be more encouraged to promote their academic work in the field of clinical nutrition, particularly during this international congress.

## Introduction

The literature on the gender bias in medicine has exploded in the last 10 years as evidenced by bibliometric data from the PubMed database. Several medical and surgical specialties have demonstrated a gender difference in the authorship of scientific publications. Women were systematically under-represented as authors, even more so as first or senior authors (1–3). This gender gap was also found in the composition of editorial boards even when women’s contribution to publishing was increasing (4, 5). Professional social networks, which offer exceptional scientific exposition, are not immune to this trend (6).

Another way to promote scholarly work and positively affect academic promotions is to participate in international conferences. International congresses are privileged spaces for disseminating one’s scientific production on a large scale *via* poster displays and oral communication sessions, where interactivity exposes the speaker all the more. However, here too, the visibility of women remains lower than that of men.

In some surgical specialties, known to be more embraced by men, it is not surprising to observe a male predominance. However, even when the proportion of women invited to congresses was proportional to women societies members, several authors have showed that women were significantly less likely to be podium presenters, particularly in plenary roles, and to receive awards or deliver invited lectures (7–9). The trend is similar in medical specialties. Assessing women’s participation at five national and international Critical Care conferences over a 7-period years, Mehta et al. (10) found that male speakers outnumbered female speakers. Kalejta and Palmenberg. explored four prominent international Virology conferences over a 35-period years. All of them showed a clear male dominance in speakers’ programmes and in session chairs (11). Even in a specialty such as Endocrinology, which has one of the highest proportion of female specialists and trainees, the trend is remarkable with more male than female speakers and a female under-representation in more prestigious roles of plenary speakers and society council members (12).

Because no other study has done so, we aimed to assess the trend of women’s visibility at the European Society for Clinical Nutrition and Metabolism (ESPEN) congress, one of the largest international congresses in the field of clinical nutrition.

## Materials and methods

### Study design and population

This retrospective study did not involve human participants and was therefore exempted from institutional ethics board review in accordance with French laws (13).

We aimed to assess women’s visibility at the ESPEN congress, in a 10-year period from 2011 to 2021 by analyzing 3 congresses: the 2011 congress, the 2021 congress and an arbitrary intermediate congress. However, the COVID-19 pandemic has deeply changed the meetings format leading to 100% virtual or hybrid congresses. To avoid potential biases related to these different modalities, we decided to exclude the 2020 and 2021 congresses from our analysis, and focused on data obtained from three ESPEN congresses, in 2011, 2015, and 2019.

For each annual ESPEN congress, a specific website reported all the information regarding the agenda, i.e., https://2019.espencongress.com in 2019: days, hours, rooms, speakers, and moderators’ identities, countries of origin, type and topic of session (oral communications, plenary sessions, laboratory symposia, educational sessions, etc.).

For the record, the moderator is the person who leads the session: he or she introduces the speakers, ensures that the allotted speaking time is respected, gives overall coherence to the speeches and acts as a link between the speakers and the audience. Being chosen as a moderator confirms the position of an expert in the field and therefore the recognition of peers. The speaker is the person who presents his or her work orally and interacts directly with the audience. Being invited as a speaker allows to present one’s work in front of a large audience of peers. This is all the more honorable as the session is specific (Lecture, Opening Ceremony.).

In this study, the following sessions (grouped under the term “major oral sessions”) were considered to provide high visibility:

– “Oral communication sessions” called “free scientific presentations,” “scientific sessions,” and “oral communications” during the different congresses,
– “Specific conferences” including “Opening ceremony,” “Sir David Cuthbertson Lecture,” “Arvid Wretlind Lecture,” “Clinical Nutrition symposium,” “Best abstracts sessions,” “GLIM session (Global Leadership Initiative on Malnutrition),” and “ESPEN Best Abstracts session,”

Industry-led symposia were also considered to be highly visible sessions as they were attended by speakers and moderators chosen by the industry for their high reputation in their field of expertise.

We also included data from educational sessions such as “educational session,” “case presentation,” “ESPEN Guidelines,” and “LLL courses.” Although perhaps less prestigious, they can provide a significant audience for less experienced speakers.

We excluded poster sessions due to the lack of available data.

### Objectives

The main objective was to describe the evolution of the proportion of women speakers at the ESPEN congress between 2011 and 2019. The primary endpoint was the percentage of women speakers in major oral sessions (oral communication sessions and specific conferences) at the 2011, 2015, and 2019 ESPEN congresses.

The secondary objectives were to describe the evolution of the representation of women in other high-visibility positions during the same period (moderators in major oral sessions, speakers and moderators in industry-led symposia), and their countries of origin.

### Data extraction

We manually searched the archives of the programmes of the three congresses (2011, 2015, and 2019), in the websites. Speaker’s gender was identified using the initial of his or her first name (or first names), surname and country of origin. The search sequence was cascaded until the correct speaker was found:

– The primary search was carried out *via* the internet search engine Google: [Initial(s) of first name(s) Last Name] + [MD nutrition] for “Medical Doctor Nutrition” because we assumed that the majority of speakers at the congress would be doctors. Example: “M Richardson MD Nutrition.” The primary search was considered unsuccessful if: the first name did not allow for easy gender attribution, there was no photograph, there were homonyms and the search area did not seem to be obviously related to the field of nutrition.
– If the primary search was unsuccessful, we added a keyword to the search: [ESPEN] and/or a keyword from the theme of the oral presentation and/or the country. Example: “M Richardson MD Nutrition ESPEN and/or gut microbiota and/or USA.”
– If the search was again unsuccessful, a further search was carried out on ResearchGate and/or PubMed with a keyword from the theme of the oral presentation + Last Name [author].
– Finally, if this last search was unsuccessful, then the speaker or moderator was classified as “non-gendered.”

The names of the countries of origin were easily recognizable by their English initials or acronyms.

### Statistical analysis

Qualitative variables are presented as percentages (n, absolute values) and compared with a Fisher’s exact test. All statistical tests were 2-sided. The significance level was 0.05 when two groups were compared to each other and 0.025 when groups were compared twice. All statistics and graphs were produced using GraphPad PRISM software (version 8.0.2; GraphPad Software).

## Results

### General characteristics of the three European Society for Clinical Nutrition and Metabolism congresses

The 2011 congress was held in Gothenburg (Sweden) under the presidency of Professor Ingvar Bosaeus; 192 moderators and/or speakers attended 6 symposia sessions, 5 specific conferences and 24 oral communication sessions. Up to 23 countries were represented.

The 2015 congress was held in Lisbon (Portugal) under the presidency of Professor Maria Cravo; 228 moderators and/or speakers attended 6 specific conferences and 36 oral communication sessions. Data for industry-led symposia were not available. Up to 29 countries were represented.

The 2019 congress was held in Krakow (Poland) under the presidency of Professor Stanislaw Klek; 231 moderators and/or speakers attended 9 symposia sessions, 7 specific conferences and 23 oral communication sessions. Up to 31 countries were represented.

### Evolution of the presence of women in high-visibility positions

Each speaker and moderator was categorized as woman, man or non-gendered. Across all sessions, non-gendered subjects represented, respectively: in 2011, 2% (1/61) of moderators, 8% (11/131) of speakers, or 6% (12/192) of all; in 2015, 9% (7/79) of moderators, 8% (12/149) of speakers, or 8% (19/228) of all; in 2019, 1% (1/71) of moderators, 12% (20/160) of speakers, or 9% (21/231) of all. Among the 52 non-gendered subjects, 12 (23%) were from Asia (Japan, China, and Korea) and 11 (21%) from Portugal.

**FIGURE 1:**
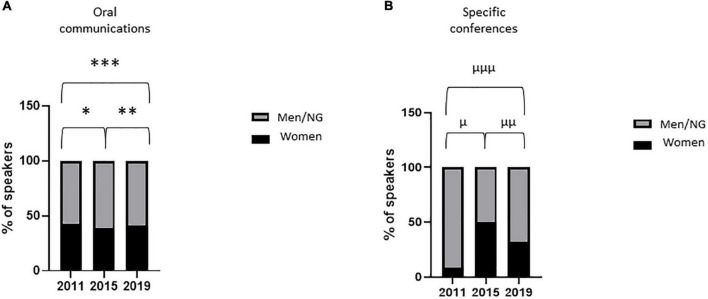
Women speakers in major oral sessions. Proportion of women speakers in major oral sessions [**(A)** oral communications; **(B)** specific conferences] during the three congresses. Variables are presented as percentages. After contingency analysis, variables were compared by Fisher’s exact test. A *p*-value < 0.025 was considered significant. *p = 0.51; ^**^p = 0.70; ^***^p = 0.89; ^μ^*p* = 0.003; ^μμ^*p* = 0.46; ^μμμ^*p* = 0.21. NG, non-gendered.

**FIGURE 2:**
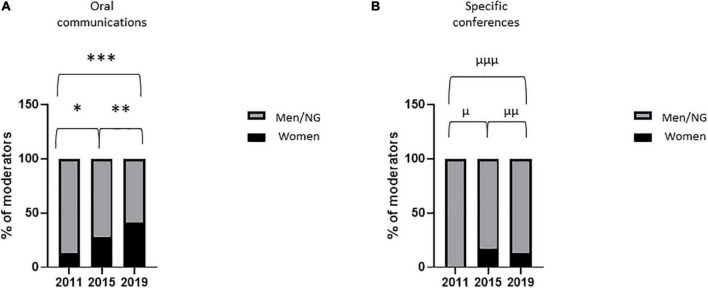
Women moderators in major oral sessions. Proportion of women moderators in major oral sessions [**(A)** oral communications; **(B)** specific conferences] during the three congresses. Variables are presented as percentages. After contingency analysis, variables were compared by Fisher’s exact test. A *p*-value < 0.025 was considered significant. *p = 0.07; ^**^p = 0.16; ^***^p = 0.004; ^μ^*p* = 0.48; ^μμ^*p* > 0.99; ^μμμ^*p* = 0.50. NG, non-gendered.

**FIGURE 3:**
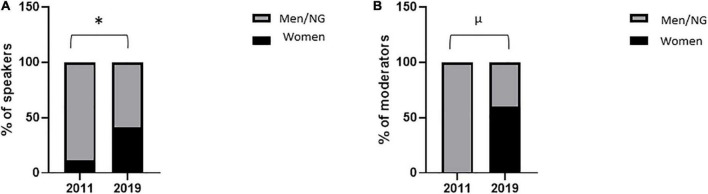
Women speakers and moderators in symposia. Proportion of women speakers **(A)** and moderators **(B)** in industry-led symposia during the 2011 and 2019 congresses. Variables are presented as percentages. After contingency analysis, variables were compared by Fisher’s exact test. A *p*-value < 0.05 was considered significant. *p = 0.05; ^μ^*p* = 0.03. NG, non-gendered.

The percentage of women speakers in oral communication sessions remained stable between 2011 and 2019 [43% (43/100) vs. 41% (46/111); *p* = 0.89] (Figure 1A) and that in specific conferences peaked in 2015 at 50% (6/12) (Figure 1B). The percentage of women moderators in oral communication sessions increased between 2011 and 2019 [13% (6/45) vs. 41% (19/46); *p* = 0.004] (Figure 2A) and that in specific conferences remained stable between 2011 and 2019 [0% (0/10) vs. 13% (2/15); *p* = 0.50] (Figure 2B). The percentage of women speakers and moderators in industry-led symposia significantly increased between 2011 and 2019 [11% (2/18) vs. 41% (11/27); *p* = 0.05; 0% (0/6) vs. 60% (6/10); *p* = 0.03, respectively] (Figures 3A,B).

**FIGURE 4:**
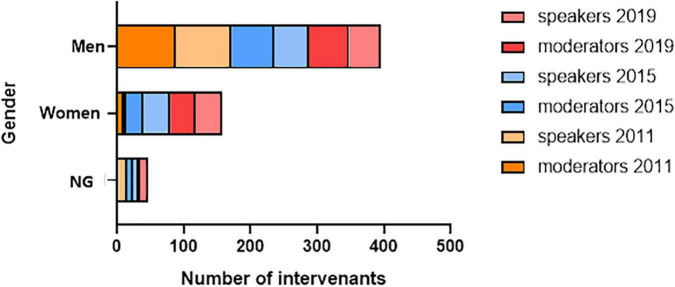
Evolution of gender during the three congresses. Distribution of gender for all types of sessions (major oral sessions and industry-led symposia) by congress year and by position (speakers and moderators). Variables are presented as absolute values. NG, non-gendered.

**FIGURE 5:**
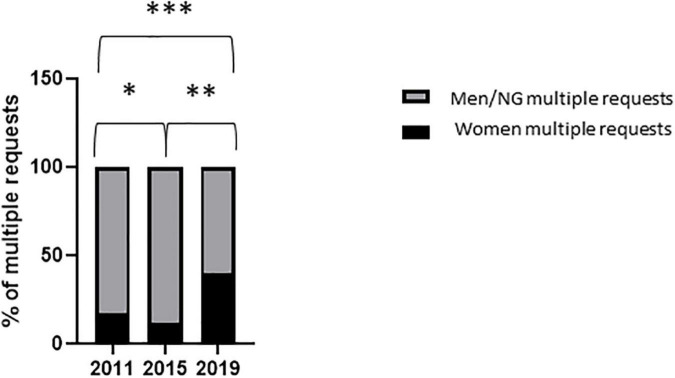
Requests for multiple interventions to women. Proportion of women solicited at least twice for all type of sessions (major oral sessions and industry-led symposia) and all positions (speakers and moderators) during the three congresses. Variables are presented as percentages. After contingency analysis, variables were compared by Fisher’s exact test. A *p*-value < 0.025 was considered significant. *p = 0.68; ^**^p = 0.01; ^***^p = 0.12. NG, non-gendered.

Men were over-represented across all congresses, positions and session types (Figure 4). The percentage of women who were solicited at least twice during the same session, whatever their position (moderator or speaker) and whatever the type of session (major oral sessions and symposia), increased significantly between 2015 and 2019 [12% (4/34) vs. 40% (14/35); *p* = 0.01; Figure 5].

### Countries of origin

**FIGURE 6:**
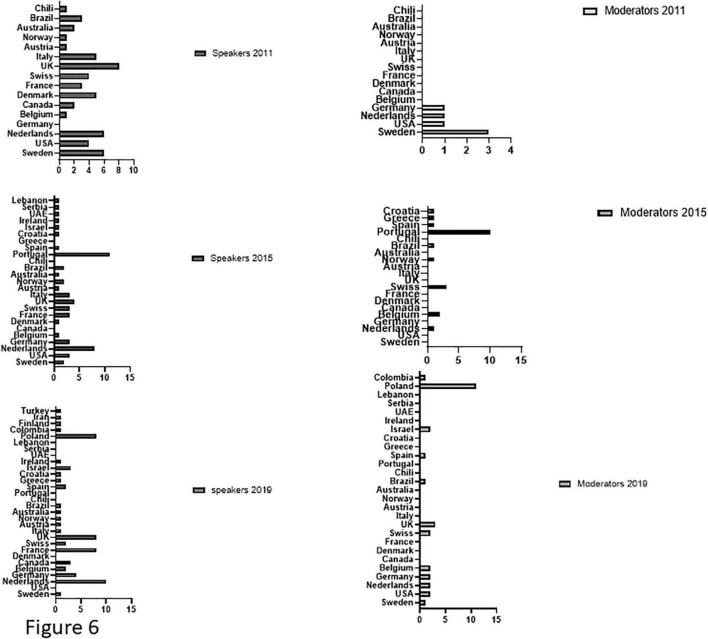
Countries of origin. Distribution of women participants (moderators and speakers) by country of origin for each of the 3 congresses. Variables are presented as absolute values.

At all three congresses, women from the host countries were over-represented as moderators compared to women from other countries: in 2011, Swedish women represented 50% (3/6) of the moderators; in 2015, Portuguese women represented 48% (10/21) of the moderators and in 2019, Polish women represented 37% (11/30) of the moderators. On the other hand, the nationals from the host countries were not necessarily the most represented among the speakers: in 2011, English women accounted for 15% (8/52) of the speakers, followed by 12% (6/52) from Sweden. In 2015, Portuguese women were in the lead at 26% (11/42) and in 2019, Polish women were equal at 13% (8/63) with English and French women, but behind the 16% (10/63) of women speakers from the Nederland (Figure 6).

### Data specific to educational sessions

At each of the 3 congresses, educational sessions were offered: 14 in 2011, 11 in 2015 and 24 in 2019. Unlike major sessions where moderators or speakers were all physicians, the educational sessions offered a platform to other non-physician professionals: out of the 252 speakers in these sessions during the 3 congresses, there were 20 Registered Dieticians, 14 Research Nurses, 1 physiotherapist, 4 scientists, and 6 pharmacists; 27 of them were women.

**FIGURE 7:**
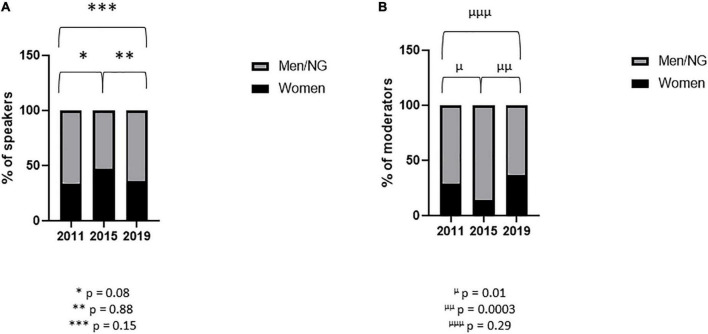
Women speakers and moderators in educational sessions. Proportion of women speakers **(A)** and moderators **(B)** in educational sessions during the three congresses. Variables are presented as percentages. After contingency analysis, variables were compared by Fisher’s exact test. A *p*-value < 0.025 was considered significant. *p = 0.08; ^**^p = 0.88; ^***^p = 0.15; ^μ^*p* = 0.01; ^μμ^*p* = 0.0003; ^μμμ^*p* = 0.29. NG, non-gendered.

The percentage of women speakers in educational sessions remained stable between 2011 and 2019 [34% (19/56) vs. 36% (30/84); *p* = 0.88] (Figure 7A). The percentage of women moderators in educational sessions also remained stable during the period with a marked under-representation of women in 2015 (Figure 7B).

## Discussion

This retrospective study is the first one showing the women’s visibility in clinical nutrition congresses, in an 8-period years by analyzing three ESPEN congresses. We have showed that in oral communication sessions, the percentage of women speakers remained stable around 40% between 2011 and 2019 while the percentage of women moderators had significantly increased. In specific conferences, including prestigious lectures, the percentage of women speakers significantly peaked in 2015 at 50% while the percentage of women moderators remained low under 13% between 2011 and 2019. Moreover, the percentage of women speakers in industry symposiums had also increased. Despite these encouraging trends, men remained over-represented and over-solicited across all ESPEN congresses, positions and sessions’ types.

### Under-representation of women

Quantify the proportion of women physicians whose research and work are focused on nutrition is complex. Indeed, clinical nutrition is a translational specialty that can be integrated in many disciplines. The ESPEN congresses gather both medical physicians (nutritionists, gastroenterologists, anesthetists, or intensivists), non-medical health professionals (dieticians, nurses) and fundamental scientists. Given the feminization of medicine, one could imagine that the representation of women in the field of Nutrition is increasing (14). However, there seems to be a gap between the demographic reality and the representation of women in congresses. In this study, the male predominance persisted during the period studied. Moreover, men who were already more represented were also more solicited in high-visibility positions. This situation has been well described in many specialties: the number of women physicians was increasing but leadership positions (department chairs, congress moderators or main authors in scientific publication) remained inaccessible to them (15, 16). In a cross-sectional study on 27 US medical specialty conferences from 2013 to 2017, while the representation of women speakers improved, the representation of women among high-visibility positions (keynote speakers, plenary speakers, and invited lectureships) was variable including zero levels some years (17). This negative trend has been partly explained by Sardelis and Drew who reported that the absence of women role models does not encourage young women to progress and share their academic work. These young women, without female mentors, may be apprehensive about exposing themselves at conferences where male experts predominate, leaving more place to young men who are inspired by these older men (18).

Educational sessions have been included separately in the analysis because they may be considered less prestigious in terms of visibility than other sessions due to a smaller audience. However, as these sessions are less renowned, they may offer the possibility for younger authors to present their work and therefore possibly for younger women, given the increase in the proportion of female health students (19). Unfortunately, we did not report the age of the speakers and moderators, because this data was not available in the websites. It would be also interesting to assess the evolution of women’s representation according to the age. In all cases, it is clear from the results of this study that even in these educational sessions, where some of the more feminized professions (dieticians, nurses) were a little more prominent, men are still over-represented at the expense of women.

In this study women were over-represented as speakers or moderators in industry-led symposia. We do not have public data to confirm this trend. Nevertheless, in other industrial sectors, some companies have implemented a real parity policy while others practice “feminism washing” (a strategy that consists for a company in putting forward a feminist discourse or highlighting women to improve its public image without really changing its internal practices) (20, 21).

### Of parity in congresses

International conference presentations increase speakers’ reputation and visibility, by displaying scientific production through a large audience. The first informal ESPEN meeting was held in Stockholm in 1979. The following meetings, annually organized early September in a different location in Europe, were progressively successful in terms of number of participants and industrial exhibitions. Between 2011 and 2019, the number of ESPEN members has increased from 2,600 to 3,500. Since 2015, the ESPEN congress is attracting more than 3,000 attendees, from Europe but not only, and the number of abstracts submissions reached more than 1,000 submissions in 2018 (22). During the three congresses studied, about thirty countries were represented by women speakers, which prove the international influence of those congresses and their attractiveness. Moreover, being an organizing country is a way of bringing its national scientists to the fore. We have indeed showed that women from the host countries were over-represented as moderators compared to women from other countries even this was not as consistent for women speakers. Only the 2015 ESPEN congress had a woman president. We do not have enough data to discuss the influence of the congress presidency on the parity of speakers, but a significant positive relationship has been highlighted between the number of women organizing a symposium and the number of women speaking during it (8, 18). Assessing gender diversity of orthopedic societies meetings, Gerull et al. (23) also found a positive correlation between the proportion of women in society leadership roles and the proportion of women speaking roles. Zaza et al. (24) confirmed this trend by showing that the feminization of the program committees of several surgery conferences positively correlated with speaker diversity. Between 1980 and 2022, ESPEN has been chaired (presidency of the Executive Committee) only by men. Between 1979 and 2019, 41 ESPEN congresses have been held annually throughout Europe. Only 4 congresses have been chaired by women (2004 in Lisbon, 2012 in Barcelona, 2015 in Lisbon, and 2018 in Madrid); it is notable that all these women presidents were from the Iberian Peninsula (22). To promote gender equality and to achieve parity at conferences, Martin (25) proposed ten rules including: a gender balance objective consistent with the membership, a stated gender speaker policy, a gender balanced organizing committee, a list of potential women, senior, mid-career or younger, who could benefit from the exposure, family-friendly facilities to support young parents, especially young mothers, and a strong determination to make things happen. To our knowledge, there is no mention in the ESPEN privacy policy of a particular interest in gender balance. Nor is there any information about the gender distribution of the membership. In 2022, a man chairs the Executive Committee, which is made up of 1 woman for 4 members, all of whom are physicians; a woman chairs the Scientific Committee, which is made up of 2 women for 6 members, all of whom are physicians; and a woman chairs the Education and Clinical Practice Committee, which is made up of 5 women (including a nurse, a pharmacist, and a dietitian) for 7 members. The coordination of the Guidelines is devolved to 3 men. It can be assumed that the under-representation of women in key positions influences the under-visibility of women during congresses.

At the end of this study, we strongly encourage the ESPEN to publish after each congress, for example in the special issue of the revue *Clinical Nutrition* reporting the most important abstracts, an “epidemiological” summary including: the complete composition of the organizing committee with full names and gender, the number of visitors, the number of participants, the number of presentations by type of session, and a classification by gender and age group of the various participants according to their role during the congress. This transparency would go hand in hand with a real gender balance policy.

### Limitations

Despite interesting findings, there are several limitations in this study. First, although the influence of ESPEN congress is international, the majority of speakers are European. Then our results cannot be extrapolated to all clinical nutrition congresses. A broader view of women’s visibility in the field of nutrition would require the joint analysis of congresses from other continents. Moreover, the first internet search was for “Medical Doctor Nutrition.” We hypothesized that, although the congresses were open to other professions, doctors were the preferred audience. This was confirmed during the analysis: the vast majority of participants identified in the selected sessions, including the educational ones, were medical doctors, clinicians or clinical researchers. Then it is possible that some of the unidentified participants are non-physicians health professionals. Second, to remain consistent with the rest of the literature, we have discussed gender of the speakers while the analysis of the data has been based on biological sex. Sex is commonly associated with a given name, whereas gender, which is a social construct, is not reported in the scientific literature. Third, although the manual search for authors was as thorough as possible, it cannot be ruled out that some authors may have been confused with their namesakes because of diminutives or misspelling in the program archives. In addition, due to cultural differences, we were sometimes unable to identify the gender of the speakers especially from Asian or Portuguese names. The non-gendered subjects were included in the analysis to account for all speakers and were arbitrarily classified as “non-women” for comparison purposes so that only those we were sure were in the women’s group. However, as the percentage of non-gendered was relatively small, around 8%, it would have had little impact on our results if they had been included in the women’s group. Fourth, the recruitment period was not consecutive as we chose to analyze data from only three congresses. Other authors (who were interested in the visibility of women in the scientific literature) have used this methodology, which consists of comparing one-off (i.e., non-consecutive) years within a given period to see a trend emerge (3, 26). A consecutive 8-year period analysis would probably have been more refined, without necessarily changing the trend considerably. Finally, we have excluded the 2020 and 2021 congresses, which had a virtual or hybrid format, to avoid bias. Martin (25) pointed out that it could be complicated for parents, and in particular mothers, to come to congresses because of the lack of suitable facilities. However, these facilities are no longer necessary in the case of a virtual format. We plan to study soon the data from the 2020–2021 congresses to see whether the possibility of presenting one’s work remotely increases the proportion of women speakers.

## Conclusion

In this study, despite encouraging trends during an 8-year period, women remained under-represented and less solicited than men did across all ESPEN congresses, positions, and sessions’ types. Speaking at international meetings increases a scientist’s exposure to his or her peers and helps to increase reputation, future collaborations and even future professional advancement. The diversity of speakers is highly beneficial to the richness of exchanges during a congress. It is therefore essential to encourage women to participate so that the speaker list really reflects the growing feminization of medical specialties, including clinical nutrition. We also strongly encourage the ESPEN to take up the issue of the gender balance through a committed policy.

## Data availability statement

The original contributions presented in this study are included in the article/supplementary material, further inquiries can be directed to the corresponding author.

## Author contributions

EO was involved in the study conception and design, acquisition of data, statistical analysis and interpretation of data, and manuscript draft. ZD was involved in interpretation of data and manuscript draft. TC was involved in statistical analysis and interpretation of data, and manuscript draft. NA was involved in the study conception and design, interpretation of data, and manuscript draft. All authors contributed to manuscript revision and read and approved the submitted version.
